# Biochemical Mechanism of Rhododendrol-Induced Leukoderma

**DOI:** 10.3390/ijms19020552

**Published:** 2018-02-12

**Authors:** Shosuke Ito, Kazumasa Wakamatsu

**Affiliations:** Department of Chemistry, Fujita Health University School of Health Sciences, 1-98 Dengakugakubo, Kutsukake-cho, Toyoake, Aichi 470-1192, Japan; kwaka@fujita-hu.ac.jp

**Keywords:** rhododendrol, 4-(4-hydroxyphenyl)-2-butanol, whitening agent: tyrosinase, melanocyte toxicity, sulfhydryl group, glutathione, cysteine, ultraviolet A, reactive oxygen species

## Abstract

*RS*-4-(4-hydroxyphenyl)-2-butanol (rhododendrol (RD))—a skin-whitening ingredient—was reported to induce leukoderma in some consumers. We have examined the biochemical basis of the RD-induced leukoderma by elucidating the metabolic fate of RD in the course of tyrosinase-catalyzed oxidation. We found that the oxidation of racemic RD by mushroom tyrosinase rapidly produces RD-quinone, which gives rise to secondary quinone products. Subsequently, we confirmed that human tyrosinase is able to oxidize both enantiomers of RD. We then showed that B16 cells exposed to RD produce high levels of RD-pheomelanin and protein-SH adducts of RD-quinone. Our recent studies showed that RD-eumelanin—an oxidation product of RD—exhibits a potent pro-oxidant activity that is enhanced by ultraviolet-A radiation. In this review, we summarize our biochemical findings on the tyrosinase-dependent metabolism of RD and related studies by other research groups. The results suggest two major mechanisms of cytotoxicity to melanocytes. One is the cytotoxicity of RD-quinone through binding with sulfhydryl proteins that leads to the inactivation of sulfhydryl enzymes and protein denaturation that leads to endoplasmic reticulum stress. The other mechanism is the pro-oxidant activity of RD-derived melanins that leads to oxidative stress resulting from the depletion of antioxidants and the generation of reactive oxygen radicals.

## 1. Introduction

**Figure 1:**
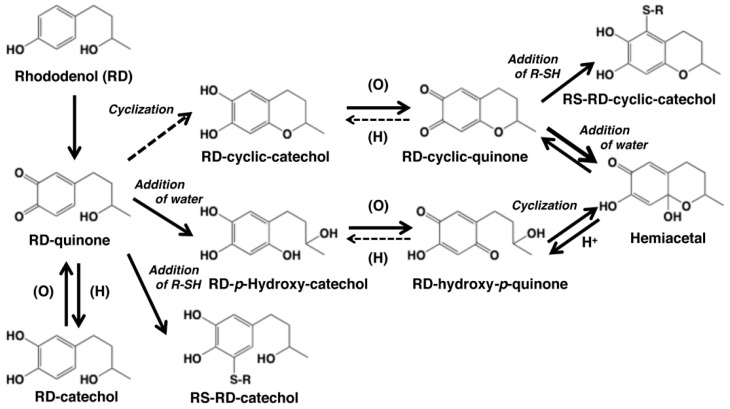
Scheme showing the tyrosinase-catalyzed oxidation of rhododendrol (RD) in the absence or presence of thiols R-SH. The oxidation of RD gives RD-quinone as an immediate product, which gives rise to RD-cyclic quinone and RD-hydroxy-*p*-quinone [9]. These quinones are produced by redox exchange of the corresponding catechols, RD-cyclic catechol and RD-*p*-hydroxycatechol, which are produced through an intramolecular cyclization of RD-quinone and the addition of a water molecule to RD-quinone, respectively. However, the feasibility of the cyclization was questioned recently by Kishida et al. [10]. RD-cyclic quinone produces RD-hydroxy-*p*-quinone instantaneously upon acidification via the hemiacetal intermediate. The reverse reaction from RD-hydroxy-*p*-quinone to RD-cyclic quinone appears to proceed rapidly at neutral pH to explain the presence of the latter quinone in the oxidation mixture. These quinones exist in an equilibrium. RD-hydroxy-*p*-quinone decays gradually through a coupling to form RD-eumelanin (see [11,12] for similar coupling reactions of hydroxy-*p*-quinones). RD-catechol can be produced through a redox exchange between RD-quinone and other catechol products. Tyrosinase-catalyzed oxidation of RD and RD-cyclic catechol in the presence of thiols (R-SH) affords the thiol adducts, RS-RD-catechol and RS-RD-cyclic catechol, respectively. Adapted from Ito et al. [9] with some modifications.

Rhododendrol (RD, Rhododenol^®^) is a naturally occurring phenolic compound found in plants such as *Acer nikoense* and *Betula platyphylla* [1,2,3,4]. RD was first isolated by Kawaguchi et al. [5] and its structure was determined to be 4-(4-hydroxyphenyl)-2-butanol (Figure 1). Since 2008, a racemic form of RD (*RS*-RD) had been added to cosmetics as a skin-whitening ingredient by a cosmetic company in Tokyo, Japan. In July 2013, cosmetics containing RD were recalled by that company because a considerable number of consumers developed leukoderma on their face, neck and hands. Among an estimated 800,000 users of RD, 19,605 subjects (as of October, 2016) reported that they developed leukoderma, accounting for 2–2.5% of users. The mechanism by which the cutaneous depigmentation is caused by RD-containing cosmetics was originally considered to be a competitive inhibition of tyrosinase by RD. However, skin biopsy samples taken from depigmented lesions of affected subjects showed fewer or no melanocytes compared with normal skin [6] and 96% of leukoderma lesions were found to occur at or close to the site of RD application [7]. On the other hand, RD did not have cytotoxic effects on keratinocytes and fibroblasts [8]. These data strongly suggested that RD had cytotoxic effects that are direct and specific to melanocytes.

In melanocytes, melanogenesis starts with the tyrosinase-catalyzed oxidation of l-tyrosine to produce dopaquinone [13,14,15,16,17,18]. Subsequent spontaneous (non-enzymatic) and enzyme-catalyzed reactions give rise to the production of eumelanin, while the intervention of l-cysteine (CySH) with dopaquinone leads ultimately to the production of pheomelanin. Tyrosinase—the key enzyme in melanogenesis—is able to oxidize, in addition to l-tyrosine, a number of phenols and catechols to form the corresponding *ortho*-quinones [15,19]. *o*-Quinones are highly reactive compounds that exert cytotoxicity through binding with sulfhydryl (thiol) enzymes and/or DNA and by producing reactive oxygen species (ROS) [20,21].

RD has a *para*-substituted alkylphenol structure. Certain exogenous *p*-substituted phenols and related compounds are known to cause contact or occupational vitiligo, which is characterized by the irreversible loss of epidermal melanocytes at both exposed and non-exposed areas [22]. Those phenolic substances, e.g., 4-*tert*-butylphenol [23], 4-methoxyphenol [24] and 4-benzyloxyphenol (monobenzone or hydroquinone monobenzyl ether) [25,26], are good substrates for tyrosinase and produce reactive *o*-quinones [27] that can bind to biologically relevant thiol compounds such as CySH and glutathione (GSH) through sulfhydryl groups. Covalent binding to bovine serum albumin (BSA) through a cysteinyl residue was confirmed for monobenzone-derived *o*-quinone [25]. In related studies to develop antimelanoma agents using tyrosinase substrates, we synthesized a number of derivatives of 4-*S*-cysteaminylphenol (4-*S*-CAP), among which *N*-propionyl-4-*S*-cysteaminylphenol (NPrCAP) was the most active in inhibiting the growth of melanoma cells in vitro and in vivo [28,29]. Our study showed that the phenol NPrCAP is activated by mushroom tyrosinase to an *o*-quinone that reacts rapidly with CySH, GSH and BSA [30]. The adduct formation with melanocytic proteins was confirmed in vitro and in vivo. In those studies, the covalent binding of BSA (or cellular thiol proteins) was confirmed by HPLC analysis of cysteinylcatechol derivatives after acid hydrolysis.

Since late 2013, we have been examining the biochemical basis of RD-induced leukoderma by elucidating the metabolic fates of RD in the course of tyrosinase-catalyzed oxidation. We reported that the oxidation of racemic RD (Figure 1) by mushroom tyrosinase rapidly produces RD-quinone, which gives rise to secondary quinone products [9]. Subsequently, we confirmed that human tyrosinase is able to oxidize both enantiomers of RD [31]. We then showed that B16 cells exposed to RD produce high levels of RD-pheomelanin and protein-SH adducts of RD-quinone [32]. Furthermore, our recent studies showed that RD-eumelanin—an oxidation product of RD—exhibits a potent pro-oxidant activity [33] that is enhanced by ultraviolet (UV)-A radiation [34]. In this review, we summarize our biochemical findings on the tyrosinase-dependent metabolism of RD and related studies by other research groups. In addition, the involvement of an immunological mechanism in the spread of depigmented lesions beyond the applied areas will be briefly addressed. A review has already been published that covers the literature on this topic until 2015 [35].

## 2. Early Events of RD Metabolism Catalyzed by Tyrosinase

RD was put on the market as a depigmenting agent because of its potent inhibition of tyrosinase activity. In fact, Sasaki et al. [36] reported that RD suppressed the tyrosinase activity of human melanocytes in culture and inhibited mushroom tyrosinase activity competitively with a *K*_i_ value of 24 μM. It was also reported that when using 3′,5′-[^3^H]-rhododendrol, RD was oxidized by mushroom tyrosinase with a *K*_m_ value of 0.27 mM, a value comparable with the *K*_m_ for l-tyrosine (*K*_m_ = 0.36 mM).

**Figure 2:**
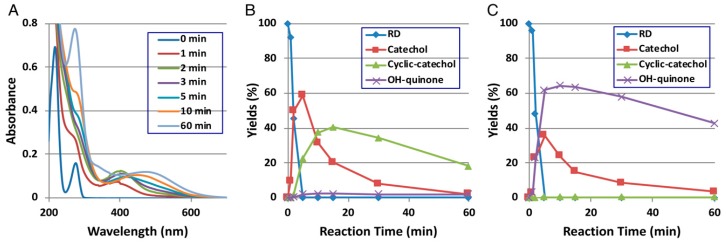
Time course of the tyrosinase-catalyzed oxidation of RD. (**A**) UV/Vis spectral changes over time at pH 5.3; (**B**) HPLC following oxidation at pH 5.3, the reaction being stopped by the addition of NaBH_4_ followed by the addition of HClO_4_; (**C**) HPLC following oxidation at pH 5.3, the reaction being stopped by the addition of HClO_4_ alone. Adapted from Ito et al. [9].

Direct evidence for the production of RD-quinone upon the tyrosinase-catalyzed oxidation of RD was reported by Ito et al. [9], who followed spectral changes in the oxidation of RD by mushroom tyrosinase. The reaction proceeded too rapidly at pH 6.8, and therefore, we slowed down the reaction by performing the oxidation at pH 5.3 (Figure 2A). Within a few minutes, an *o*-quinone having an absorption maximum near 400 nm appeared, which was replaced in 10 min by a new, distinct absorption peak near 450 nm, suggesting a conversion to secondary *o*-quinones. This absorption changed further to a spectrum having absorption maxima at 274 and 474 nm.

To identify the oxidation products, we then followed the reaction by HPLC using UV detection at 280 nm to detect both phenols and catechols. The oxidation was stopped by reduction with NaBH_4_ to convert *o*-quinones to catechols (Figure 2B). Preparative HPLC afforded two products, one of which was the expected RD-catechol and the other was found to be the RD-cyclic catechol (Figure 1). This identification led to confirmation of the structures of the oxidation products having absorption maxima near 400 nm and 460 nm as RD-quinone and RD-cyclic quinone, respectively. When the oxidation was stopped by acidification with HClO_4_, another product appeared as a major product (Figure 2C). This compound could not be isolated because of its high instability, but available evidence suggested it to be RD-hydroxy-*p*-quinone (Figure 2C). It was originally interpreted that RD-cyclic catechol and RD-*p*-hydroxycatechol arise from an intramolecular addition of the hydroxy group of RD-quinone and an addition reaction of a water molecule to RD-quinone, respectively. Interestingly, upon acidification to pH < 1, RD-cyclic quinone was rapidly converted, through a hemiacetal intermediate, to RD-hydroxy-*p*-quinone, which had absorption maxima at 270 and 380 nm at pH < 1, similar to those of 4-hydroxydopamine-2,5-quinone (262 and 382 nm).

Kishida et al. [10] investigated the cyclization reaction of RD-quinone using density functional theory-based first principles calculations and found that RD-quinone in the electroneutral structure cannot undergo cyclization, indicating a slow cyclization of RD-quinone at neutral pH. That study also showed that RD-quinone has a preference toward thiol binding rather than cyclization compared to dopaquinone. If the intramolecular addition of the hydroxy group in RD-quinone to form RD-cyclic catechol would be too slow to proceed, another mechanism for the generation of RD-cyclic quinone should be considered. If RD-*p*-hydroxycatechol would be produced much faster from RD-quinone than RD-cyclic catechol, its oxidized form, RD-hydroxy-*p*-quinone, may undergo the reverse course of reactions to form RD-cyclic quinone. RD-cyclic quinone and RD-hydroxy-*p*-quinone appear to exist in equilibrium and gradually undergo dimerization (see [10,35] for similar coupling reactions). The co-existence of RD-cyclic quinone and RD-hydroxy-*p*-quinone can explain their chemical behaviors as described above.

Thiols such as CySH and GSH have a high reactivity toward *o*-quinones [15,37]. We examined whether RD-quinone and RD-cyclic quinone are able to react with thiols (R-SH) to form R-SH adducts. This type of addition reaction, thiol binding, was confirmed for CySH, GSH and *N*-acetylcysteine [9]. In a subsequent study [32], we examined the reactivity of BSA with RD-quinone and RD-cyclic quinone. We found that both quinones bound to BSA effectively through a cysteine residue with yields of about 60%, which is much higher than the binding efficacy (<10%) of dopaquinone [38,39].

Racemic (*RS*)-RD was used as a topical skin-whitening agent. We then examined whether human tyrosinase is able to oxidize either or both of the enantiomers of RD [31]. Using a chiral HPLC column, racemic RD was resolved optically to *R*(−)-RD and *S*(+)-RD enantiomers. In the presence of a catalytic amount of l-dopa, human tyrosinase, which can oxidize l-tyrosine but not d-tyrosine, was found to oxidize both *R*(−)-RD and *S*(+)-RD to give RD-catechol and its oxidation products. *S*(+)-RD was more effectively oxidized than l-tyrosine, while *R*(-)-RD was as effective as l-tyrosine.

## 3. RD Metabolism In Vitro and In Vivo

**Figure 3:**
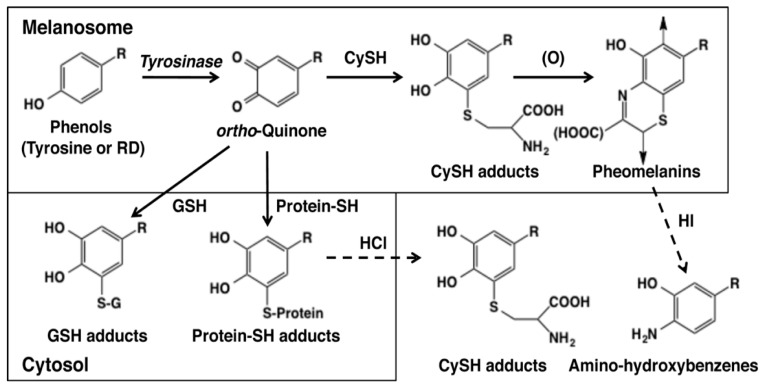
Metabolic pathways for phenols (such as RD or l-tyrosine) catalyzed by tyrosinase in melanin-producing cells [32]. The oxidation of RD or tyrosine with tyrosinase gives RD-quinone or dopaquinone as an immediate product in melanosomes. These quinones bind CySH to give CyS-RD-catechol or CyS-Dopa, respectively. They are oxidized to RD-pheomelanin and Dopa-pheomelanin. RD-eumelanin and Dopa-eumelanin are also produced in melanosomes [15]. RD-pheomelanin and Dopa-pheomelanin can be analyzed as amino-hydroxybutylbenzene and amino-hydroxyphenylalanine, respectively, after HI hydrolysis [32,44]. When leaked to the cytosol, these quinones bind to GSH to give GS-RD-catechol or GS-Dopa, respectively. CyS-catechols and GS-catechols can be analyzed as such by HPLC. Quinones that have escaped from binding to CySH or GSH react with protein-SH to form protein-*S*-RD-catechol or protein-*S*-Dopa. These protein adducts can be analyzed as CyS-RD-catechol or CyS-Dopa, respectively, after HCl hydrolysis [32,38,39].

As the early events of RD metabolism by tyrosinase-catalyzed oxidation were clarified, we next examined whether those reactions actually occur in vitro [32]. Figure 3 depicts possible metabolic products in the tyrosinase-catalyzed reactions of l-tyrosine and RD. *o*-Quinones are produced at the beginning which then bind to CySH that is present abundantly in melanosomes [15,40]. The CySH adducts are oxidized to form pheomelanic pigments (natural pheomelanin and RD-pheomelanin) via benzothiazine intermediates. Pheomelanin is known to exert cytotoxicity to melanocytes through the production of ROS and the depletion of antioxidants [41,42,43]. The amounts of pheomelanic pigments can be analyzed as amino-hydroxybenzene derivatives produced upon hydroiodic acid hydrolysis of pheomelanic pigments [44]. *o*-Quinones that are produced excessively in melanosomes leak to the cytosol and react with GSH that is present there at high levels to produce GSH adducts. This process should result in the consumption of GSH, possibly leading to oxidative stress. On the other hand, this GSH binding would be a detoxifying process against toxic *o*-quinones. *o*-Quinones that have escaped from binding to the small thiols CySH and GSH would then bind to cellular proteins through the thiol group of cysteine residues. This protein binding is considered one of the most important mechanisms of cytotoxicity. The extents of protein binding can be analyzed as CyS-adducts, such as CyS-Dopa or CyS-RD-catechol, after HCl hydrolysis [32,38,39].

Based on the above background, we examined the metabolism of RD in B16F1 mouse melanoma cells in vitro in a collaboration with Toshiharu Yamashita of the Sapporo Medical University [32]. Melanoma cells were exposed to 0.3 mM or 0.5 mM RD (toxic concentration) for three days and metabolites were analyzed by HPLC. The results are summarized in Table 1. Eumelanin levels were reduced to one-eighth that of the control, which accounts for the hypopigmentation of melanocytes exposed to RD. RD-pheomelanin was detected in the RD-exposed cells. Unfortunately, we could not develop a method to detect RD-eumelanin. However, the detection of RD-pheomelanin suggests the production of RD-eumelanin, because the normal course of melanogenesis is known to always produce both eumelanin and pheomelanin (the concept of mixed melanogenesis) [15]. CyS-RD-catechol, an adduct of CySH to RD-quinone, was detected in addition to CyS-Dopa. GS-RD-catechol, an adduct of GSH to RD-quinone, was produced at two-fold greater levels than GS-Dopa, suggesting a high level of excretion of RD-quinone to the cytosol. Adducts to RD-cyclic quinone were not detected. RD-quinone also bound effectively to proteins through the SH group. We observed 20- to 30-fold greater levels of binding of RD-quinone compared to dopaquinone. This high level of protein binding suggests the possibility that the inactivation of SH enzymes and the denaturation of SH proteins are the major mechanism of cytotoxicity of RD-quinone. Lastly, we expected that the cellular levels of GSH and CySH would be much reduced due to binding to RD-quinone. Surprisingly, the levels of GSH and CySH were elevated by exposure to RD roughly two-fold and 10- to 15-fold compared to the control, respectively. This suggests that melanoma cells that have survived the cytotoxicity of RD have a much higher activity of the detoxifying mechanism. This issue will be discussed later.

In a collaborative study with Chikako Nishigori of Kobe University [45], we examined the metabolism of RD in human epidermal melanocytes exposed to UVB irradiation. Higher levels of CyS-RDC and RD-pheomelanin were found in melanocytes treated with RD and UVB compared to the RD only-treated cells.

RD-catechol was detected in human melanocytes with high levels of tyrosinase activity [46]. In our in vitro study using B16 melanoma cells [32], RD-catechol was not detected in cells but was detected in the culture medium.

In addition to the in vitro study, we examined the metabolism of RD in vivo in collaboration with Tamio Suzuki’s group at Yamagata University [47]. In this study, hairless hk14-SCF transgenic mice, which have melanocytes distributed in the epidermis, were treated daily with 30% RD for 28 days. Histological examination indicated a decrease of epidermal melanocytes as early as day 7 and depigmentation in the RD-treated sites appeared on day 14. Eumelanin content was decreased to a 60% level on day 15 while pheomelanin content was increased to some extent. Adducts of RD-quinone to GSH, CySH and protein-SH were also detected in addition to RD-catechol. An earlier in vivo study using brown or black guinea pigs also showed marked decreases of epidermal melanocytes [48].

## 4. Late Events of RD Metabolism in Relation to the Pro-Oxidant Activity

**Figure 4:**
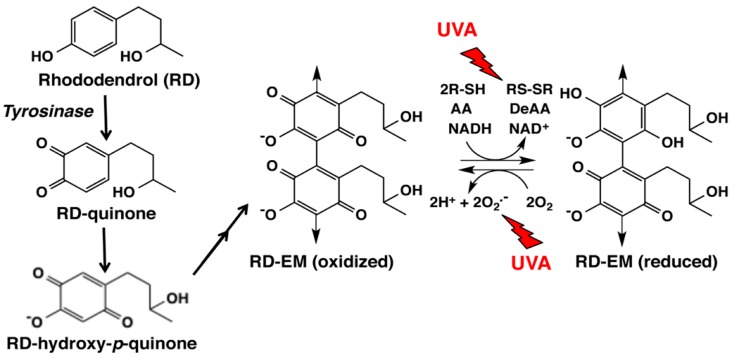
Tyrosinase-catalyzed oxidation of RD giving rise to RD-eumelanin (RD-EM). Our previous study [9] showed that the immediate product is RD-quinone, which undergoes the addition of a water molecule and is oxidized to form RD-hydroxy-*p*-quinone. RD-hydroxy-*p*-quinone gradually dimerizes to form RD-EM [9,11,12]. The oxidized form of RD-EM is able to oxidize thiols (R-SH), ascorbic acid (AA) and NADH to disulfides (RS-SR), dehydroascorbic acid (DeAA) and NAD^+^, respectively, while the reduced form of RD-EM is able to reduce molecular oxygen producing superoxide radicals. It is likely that RD-EM is a mixture of dimers and tetramers [33]. Our latest study showed that the pro-oxidant activity of RD-EM is enhanced by UVA radiation [34].

In a recent study, we examined changes of GSH and CySH in B16 melanoma cells exposed to RD for up to 24 h [33]. CySH, but not GSH, was found to decrease during 0.5 to 3 h exposure, due to oxidation to cystine. This selective depletion of CySH may be explained by assuming that this reaction takes place in melanosomes [40]. This pro-oxidant activity was then compared for synthetic melanins prepared from RD or Dopa in the absence or presence of CySH by oxidation with mushroom tyrosinase. RD-eumelanin—a mixture of dimeric and tetrameric RD-hydroxy-*p*-quinone; Figure 4—was found to exert a pro-oxidant activity as potent as Dopa-pheomelanin (Table 2). GSH, CySH, ascorbic acid and NADH were oxidized by RD-eumelanin with a concomitant production of H_2_O_2_ (Figure 4). RD-eumelanin is thus redox-active, possessing both oxidizing and reducing activities. This redox property has been known for many years for melanins [49,50], and a recent study by Kim et al. [51] demonstrated that both pheomelanin and eumelanin are redox-active and that they can rapidly and repeatedly redox-cycle between the oxidized and reduced states.

In our latest study [34], we examined whether this pro-oxidant activity of RD-eumelanin is enhanced by ultraviolet A (UVA) radiation because most RD-induced leukoderma lesions were found in sun-exposed areas [7]. That study also showed that RD-induced leukoderma occurred more frequently in the summer months compared to the spring and autumn months [7]. We found that exposure to a physiological level of UVA (3.5 mW/cm^2^) [52] induced two- to four-fold increases in the rates of oxidation of GSH, CySH, ascorbic acid and NADH. This oxidation is oxygen-dependent and is accompanied by the production of H_2_O_2_. Based on these findings, we propose that RD-eumelanin induces cytotoxicity through its potent pro-oxidant activity that is enhanced by UVA radiation. This study thus suggests UVA radiation as an additional causative factor of RD-induced leukoderma, although the frequent occurrence of RD-induced leukoderma in summer months can be due to the frequent use of RD-containing cosmetics by most of users in sun-exposed area in summer.

## 5. Melanocyte Toxicity of RD and the Detoxifying Mechanism

**Figure 5:**
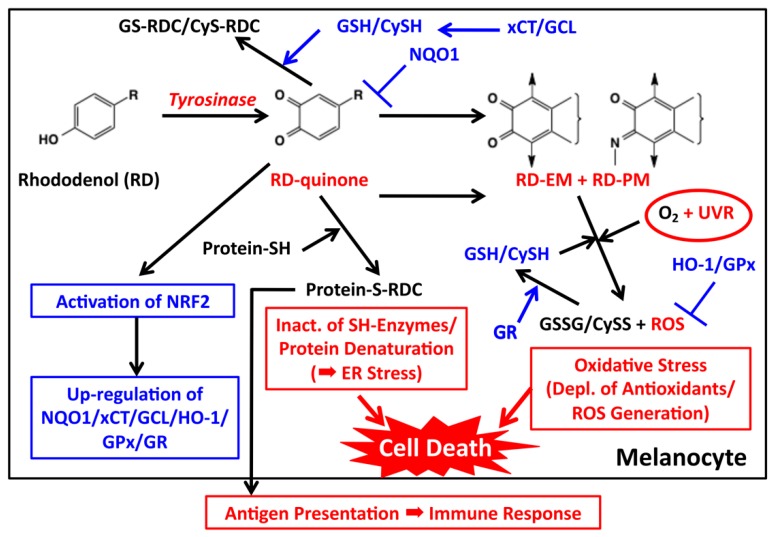
Proposed biochemical mechanism of RD-induced leukoderma. Toxic events are shown in red while detoxifying events are shown in blue. Tyrosinase catalyzes the oxidation of RD to form two toxic metabolites, RD-quinone and RD-melanin. RD-quinone reacts with CySH and GSH to give the adducts, CyS-RD-catechol (CyS-RDC) and GS-RD-catechol (GS-RDC). RD-quinone also reacts with protein-SH to give the protein adduct, protein-*S*-RD-catechol (protein-*S*-RDC). This reaction leads to the inactivation of SH enzymes and the unfolding of SH proteins, which lead to cytotoxicity and ER stress, respectively. This protein binding may produce antigens, which lead eventually to an immune response. RD-quinone is then further metabolized to RD-eumelanin and RD-pheomelanin. These melanins, especially RD-eumelanin, exert a potent pro-oxidant activity oxidizing antioxidants and producing ROS. This pro-oxidant activity can be enhanced by UVA radiation. UVB might also be involved in this process. These reactions lead to oxidative stress.

Based on the above findings on the early and late events of RD metabolism, we propose the mechanism for the melanocyte toxicity of RD and the detoxifying mechanism against RD as summarized in Figure 5.

### 5.1. Roles of Tyrosinase

Tyrosinase is able to oxidize a great number of phenols and catechols to form *o*-quinones. We have shown that mushroom tyrosinase oxidizes not only RD but also other leukoderma-inducing phenols, such as raspberry ketone [53], 4-methoxyphenol, 4-benzyloxyphenol, 4-*tert*-butylphenol and 4-*tert*-butylcatechol, to form the corresponding *o*-quinones [27]. It is apparent that tyrosinase initiates the first step toward the cytotoxicity of RD. In fact, Sasaki et al. [36] showed that treatment with phenylthiourea, a chelator of the copper ions necessary for tyrosinase activity, attenuated RD-induced cytotoxicity in a dose-dependent manner. Similarly, the depletion of tyrosinase by a siRNA against tyrosinase mRNA almost completely rescued melanocytes from damage by RD at concentrations as high as three millimolars. Kasamatsu et al. [46] used 13 different lines of human normal melanocytes with different levels of tyrosinase activity to show that melanocyte damage was related to tyrosinase activity at a certain threshold.

Other studies also indicated a role for tyrosinase in the cytotoxicity of RD to melanocytes. In the in vivo study by Abe et al. [47] (see above), albino mice that lack tyrosinase activity did not show a change in the number of melanocytes when treated with RD for 14 days.

### 5.2. Cytotoxicity of o-Quinones (RD-Quinone and RD-Cyclic Quinone)

*o*-Quinones are potent cytotoxic compounds. The cytotoxicity of *o*-quinones appears to be correlated to the efficacy of binding to cellular proteins. The protein binding leads to the inactivation of SH-enzymes that are essential for cell proliferation and survival such as DNA polymerase [20,21]. The cellular site(s) of adduct formation between RD-quinone and thiol proteins needs to be discussed as it is often mentioned that it is melanosomal proteins that bind to *o*-quinones. However, the detection of GS-RD-catechol in addition to CyS-RD-catechol is a good indication that a considerable portion of RD-quinone is able to escape from melanosomes to the cytosol where it binds to GSH (Figure 3) [32]. Thus, considering the much lower reactivity of protein-SH compared to CySH and GSH [38,39], most, if not all, of the addition reaction of protein-SH may take place in the cytosol.

How the binding of RD-quinone (2) to protein-SH induces cytotoxicity to melanocytes is a matter of conjecture. A number of studies have shown that RD induces endoplasmic reticulum (ER) stress and the apoptosis that follows [8,36,45,54,55]. Sasaki et al. [36] found that the gene expression level of CCAAT-enhancer-binding protein homologous protein (CHOP), a transcription factor with a major role in unfolded protein response (UPR)-induced cell death, was up-regulated in melanocytes exposed to RD and this up-regulation was tyrosinase-dependent. Also, the release of IL-8 to the culture medium was found to be up-regulated in a tyrosinase-dependent manner. This tyrosinase-dependent cell death induced by RD was found to be apoptotic, as revealed by the increased level of cleaved caspase-3. Lee et al. [54] also reported caspase-3 activation by RD, which is increased by UVB radiation. Arase et al. [55] reported the augmentation of UVB-induced apoptosis of melanocytes exposed to RD and the suppression of NF-κB activation by TNFα that depends on tyrosinase activity. Goto et al. [45] reported that UVB radiation enhanced RD-induced cytotoxicity in melanocytes via the induction of ER stress. An in vivo study by Abe et al. [47] also suggested the involvement of ER stress using biopsy samples from RD exposed mice. In addition, Yang et al. [8] showed that the autophagy-lysosome pathway is involved in RD cytotoxicity using autophagy-deficient and autophagy-enhanced melanocytes.

However, what metabolic event actually elicits the ER stress was not addressed in those studies. One possible cause of ER stress is the accumulation of unfolded proteins [56]. Based on the high level of production of protein-SH adducts to RD-quinone (Table 1), we propose that the accumulation of proteins that are denatured through the binding to RD-quinone should lead to ER-stress and eventually to apoptosis. Other mechanisms of cytotoxicity may also be possible, such as cell damage through antioxidant depletion and/or ROS generation. These mechanisms are not mutually exclusive. This possibility will be discussed later.

If the generation of RD-quinone triggers cytotoxicity to melanocytes, its reduction to RD-catechol within the cells would suppress the cytotoxicity by RD. This was found to be the case. Okubo et al. [57] examined the effect of the forced expression of NAD(P)H quinone dehydrogenase, quinone 1 (NQO1), a major quinone-reducing enzyme in the cytosol (Figure 5) [58]. They found that treatment of B16BL6 mouse melanoma cells or normal human melanocytes with carnoic acid, a transcriptional inducer of the *NQO1* gene through activation of the transcription factor, nuclear factor E2-related factor 2 (NRF2), significantly mitigated the cytotoxicity of RD. NRF2 is a master regulator of anti-oxidative responses [59]. Okubo et al. [57] also showed that RD-induced apoptosis, as examined by the cleavage of caspase 3 and poly-ADP ribose polymerase, was strongly suppressed by carnoic acid. RD itself was found to activate the expression of NRF2 and NQO1 in B16 melanoma cells, although the effects of RD were weaker than carnoic acid.

Another possible way for melanocytes to protect themselves from the cytotoxicity of RD-quinone would be to scavenge it through binding with non-protein thiols GSH and CySH (Figure 5). GSH is a major thiol present in the cytosol while CySH is in melanosomes [40]. In fact, we detected GS-RD-catechol and CyS-RD-catechol in B16 melanoma cells exposed to RD (Table 1). In this respect, Kondo et al. [60] showed that GSH maintenance is crucial for the survival of melanocytes after exposure to RD. Pretreatment of normal human epidermal melanocytes with *N*-acetylcysteine, which leads to an increase in GSH levels, attenuated the cytotoxicity induced by RD, while pretreatment of cells with L-buthionine sulfoximine, a potent and selective inhibitor of GSH synthesis, led to an increased cytotoxicity of RD (and a decrease in GSH). A similar protective role of *N*-acetylcysteine was also reported by Kim et al. [61]. RD exposure up-regulated the mRNA expression levels of *glutamate cysteine ligase* (*GCL*), *NQO1* and *heme oxygenase 1* (*HO-1*) in a dose-dependent manner [60]. GCL is a rate-limiting enzyme of GSH synthesis [62] while HO-1 is an enzyme involved in phase II detoxification [63]. Kondo et al. [60] used an *NRF2*-specific siRNA to show that the RD-induced activation of NRF2 attenuated GSH depletion and promoted melanocyte survival. Cystine-glutamate exchanger (xCT) may act together with GCL to supply GSH (and CySH) that have been depleted through thiol binding to RD-quinone (Figure 5). xCT is a solute carrier family member 11 (SLC7A11) that transports cystine into cells [64].

The binding of GSH to RD-quinone should lead, at least transiently, to the depletion of GSH (and CySH). In fact, in human melanocytes, exposure to 0.1 mM RD for six hours significantly reduced GSH levels [60]. Contrary to our expectation, when we analyzed GSH and CySH levels after exposure of RD to melanoma cells for three days, those levels were significantly increased (Table 1) [32]. The reason for this increase in GSH has now become clear. As mentioned above, the up-regulation of GCL would contribute to the increase in GSH. In fact, GSH levels increased in a dose-dependent manner from the control levels 24 h after RD exposure through the transient decrease at six hours [60].

RD-cyclic quinone and RD-hydroxy-*p*-quinone are highly reactive quinones as evidenced by their rapid decay in the oxidation mixture (Figure 2B,C). However, we could not detect products of thiol binding with RD-cyclic quinone in B16 melanoma cells treated with RD [32].

### 5.3. Cytotoxicity of Catechols (RD-Catechol and RS-RD-Catechol)

Catechols are known to be cytotoxic to various types of cells, especially to melanocytes and melanoma cells. Two general mechanisms exert the cytotoxicity, one through the production of *o*-quinones and the other through the production of ROS during autoxidation of catechols or redox-cycling of catechols/*o*-quinones [20,65]. It may be possible that various catechols produced during the tyrosinase-catalyzed oxidation of RD augment cytotoxic effects on melanocytes. In fact, Okura et al. [66] showed that RD-catechol was approximately 10 times more toxic to B16 melanoma cells and two lines of normal human melanocytes compared to RD and that RD-cyclic catechol was even more toxic than RD-catechol. Another study also found that RD-catechol was much more toxic to human melanocytes [36].

The adduct formation of *o*-quinones with GSH may not necessarily be a detoxifying event, as some GSH-quinone adducts (such as GS-RD-catechol) retain the ability to redox cycle with the concomitant formation of ROS [67]. However, the protective effects of NQO1 and GSH in detoxifying RD-derived metabolites (as discussed in the previous section) led us to believe that it is RD-quinone rather than RD-catechol that plays a central role in the cytotoxicity to melanocytes exposed to RD.

### 5.4. Cytotoxicity by the Pro-Oxidant Activity of RD-Eumelanin and RD-Pheomelanin

Several studies have detected ROS generation in melanocytes exposed to RD. For example, Okura et al. [66] showed that RD and RD-catechol produced ROS detected both by flow cytometry and by immunostaining in B16F1 melanoma cells and human melanocytes. Nagata et al. [68] reported a greater level of ROS in B16F10 cells exposed to RD compared to HaCaT keratinocytes. Kim et al. [61] showed approximately 10-fold increased levels of ROS generation in RD-exposed B16F10 cells using a fluorescence assay. Furthermore, Goto et al. [45] reported that ROS generation was synergistically enhanced in normal human melanocytes treated with RD and UVB compared to cells treated with RD alone.

Then how can ROS be generated in RD-treated melanocytes? Natural Dopa-pheomelanin is known to produce superoxide radicals in the dark [42,43], which is accelerated by UV radiation [69]. Our recent study showed that RD-eumelanin is a pro-oxidant as active as Dopa-pheomelanin in oxidizing cellular antioxidants such as GSH, CySH, ascorbic acid and NADH with a concomitant production of H_2_O_2_ (Figure 4) [33]. This pro-oxidant activity is significantly enhanced by UVA radiation [34]. RD-pheomelanin possesses a pro-oxidant activity about one-half that of RD-eumelanin. Thus, the production of RD-eumelanin and RD-pheomelanin would lead to both the depletion of antioxidants and the generation of ROS (Figure 5). These pro-oxidant effects of RD-derived melanins can be counter-acted by various antioxidant mechanisms (Figure 5). HO-1 is involved in the survival of H_2_O_2_-exposed melanocytes and the susceptibility to vitiligo [63]. It is interesting that up-regulation of the *HO-1* gene was approximately 500-fold compared to only three-fold up-regulation of the *GCL* and *NQO1* genes [60]. In addition to NQO1, GCL and HO-1, some other antioxidant enzymes can be up-regulated by the NRF2 signaling pathway (Figure 5) [70]. Thus, glutathione peroxidase (GPx) reduces peroxides in the presence of GSH. In this process, GSH is oxidized to GSSG, which in turn is reduced back to GSH by glutathione reductase (GR) [71]. In this respect, Kim et al. [61] reported that GPx was depleted by the exposure of B16 cells to RD, whose extent was significantly attenuated by *N*-acetylcysteine. In melanocytes that are surviving the RD-induced toxicity, GSH (and CySH) acts to cope with the cytotoxicity not only through the direct scavenging of RD-quinone but also through the concerted action with GPx (and GR and HO-1) to decompose ROS, in particular H_2_O_2_ (Figure 5).

ROS generation during the tyrosinase-catalyzed oxidation of RD was studied by Miyaji et al. [72]. That study detected hydroxyl radical and singlet oxygen during the tyrosinase-catalyzed oxidation of RD using ESR trapping techniques. However, hydroxyl radicals are not directly produced but are derived from H_2_O_2_ that is produced from superoxide radicals. Certainly, more studies are necessary to clarify the tyrosinase-depended production of ROS from RD.

## 6. Immunological Mechanisms

This review is not intended to discuss in depth the immunological aspects of RD-induced leukoderma and its similarity (or dissimilarity) to vitiligo vulgaris. However, immunological reactions are certainly involved in the pathogenesis of the RD-induced leukoderma. For example, Tanemura et al. [6] reported an increase of CD4^+^ and CD8^+^ cells infiltrating the dermis of RD-induced leukoderma lesions. Fujiyama et al. [73] observed a high frequency of Melan-A-specific cytotoxic T lymphocytes in RD leukoderma patients. Nishioka et al. [74] found larger numbers of CCR4^+^ CD8^+^ cells and higher concentrations of CCL17 and CCL22. These findings may help explain the depigmentation in skin areas not directly exposed to RD and its expansion in some patients [75,76].

How do RD or RD metabolites trigger immunological responses? One possibility is the haptenation theory that was put forth to explain the molecular mechanism of monobenzone-induced skin depigmentation (Figure 5) [26,77]. Phenolic substrates as prohaptens are oxidized by tyrosinase to produce *o*-quinones, which act as haptens that covalently bind to tyrosinase or other melanosomal (or melanocytic) proteins to generate possible neo-antigens [26,30]. These neo-antigens, in turn, can trigger an immunological response cascade that leads to melanocyte loss to produce depigmentation. However, RD usually causes melanocyte loss only at the applied sites [7], suggesting that immunological responses may not play a major role in the initiation of the RD-induced cytotoxicity.

## 7. Concluding Remarks

How RD is metabolized by tyrosinase in melanocytes is summarized in Figure 1. RD produces two major toxic metabolites, RD-quinone and RD-melanins. RD-quinone is a highly reactive compound that binds to SH-proteins, leading eventually to cell death (Figure 5). RD-melanins, especially RD-eumelanin, are pro-oxidants that deplete cellular antioxidants with ROS production, leading eventually to cell death. The toxicity of RD-quinone could be prevented through binding to small thiols, GSH and CySH, or through reduction to RD-catechol by NQO1. The toxicity of RD-melanins could be prevented by a concerted action of antioxidant enzymes that are up-regulated by NRF2 (Figure 5).

Then why did only roughly two percent of RD consumers actually develop leukoderma? Figure 5 suggests several factors that lead to RD-induced cytotoxicity, including tyrosinase activity, levels of antioxidant defense mechanism controlled by NRF2 activity, ER-stress response and UV radiation. The activity of tyrosinase is determined by genetic factors [78] and hormonal factors [14,17] as well as by environmental factors, especially its delayed activation by UVB exposure [79]. In this regard, how much RD was applied to the skin by each consumer is important because rather high doses of RD were required to cause cytotoxicity in cultured melanocytes [8,32,36,46] and in an animal model [47,48]. The activity of the ER-stress response may determine whether melanocytes would survive or die [56]. In addition, autophagy may be involved in resistance to the cytotoxicity of RD [8]. Although UVB appears to augment the cytotoxicity of RD [45,54,55], how UVB interacts with RD metabolites directly or indirectly has yet to be examined. Also, the role of UVA in RD-induced cytotoxicity remains to be confirmed by in vitro studies.

## Figures and Tables

**Table 1:** Concentrations of Tyrosine- and RD-derived Metabolites in B16 Melanoma Cells Cultured for Three Days ^a^.

Metabolite ^b^	RD Not Added	RD 0.3 mM	RD 0.5 mM
Eumelanin	17 μg	1.8 μg	2.1 μg
Pheomelanin	0.71 μg	0.57 μg	0.47 μg
RD-pheomelanin	0.00 μg	0.51 μg	0.61 μg
CyS-Dopa	0.17 nmol	0.33 nmol	0.36 nmol
CyS-RD-catechol	0.00 nmol	0.09 nmol	0.15 nmol
GS-Dopa	0.24 nmol	0.08 nmol	0.12 nmol
GS-RD-catechol	0.00 nmol	0.17 nmol	0.26 nmol
Protein-*S*-Dopa	0.16 nmol	0.11 nmol	0.10 nmol
Protein-*S*-RD-catechol	0.00 nmol	2.2 nmol	3.1 nmol
GSH	25 nmol	38 nmol	59 nmol
CySH	1.3 nmol	13 nmol	20 nmol

^a^ Summarized from the data in Ito et al. [32]; ^b^ Concentrations are per one million cells. Averages are from three experiments.

**Table 2:** Oxidation of various antioxidants by synthetic melanin ^a^.

Antioxidant ^b^	Control	RD-EM	RD-PM	Dopa-EM	Dopa-PM
GSH, 60 min	0.7	67.3	29.7	33.0	62.3
CySH, 30 min	10.7	90.3	41.3	79.7	80.0
AA, 30 min	3.9	83.1	71.0	75.9	46.4
NADH, 120 min	5.7	58.7	37.7	85.5	84.8

^a^ Summarized from the data in Ito et al. [33]. Melanins were prepared by tyrosinase oxidation of precursors (RD or Dopa in the absence/presence of CySH). Oxidation was carried out with one molar equivalent of melanin (as a precursor) for the indicated time. Control contained tyrosinase alone; ^b^ Data are percent consumption of the antioxidant. Averages are from three experiments. Variations were less than five percent in most cases. Most of GSH or CySH were oxidized to GSSG or CySSCy.
